# Analysing the application of a flexible formwork pre-cast wall driving roadway along goaf in a large mining height face

**DOI:** 10.1038/s41598-024-67211-6

**Published:** 2024-07-15

**Authors:** Xiaofan Cao, Xiaoli Wang, Huihui Liu, Song Wang, Deyong Wang, Zhongping Liu

**Affiliations:** 1 School of Architecture and Civil Engineering, Xi’an University of Science and Technology, Xi’an, 710054 Shaanxi China; 2 Shaanxi Pioneer Construction Technology Co., Ltd, Xi’an, 710054 Shaanxi China; 3 Shaanxi Pioneering New Mineral Engineering Co., Ltd., Xi’an, 710054 China; 4 School of Management, Xi’an University of Science and Technology, Xi’an, 710054 Shaanxi China

**Keywords:** Large mining height working face, Flexible formwork concrete, Driving roadway along goaf, Mechanical model, Numerical simulation, Energy science and technology, Coal

## Abstract

The technology of building a retaining roadway along goaf or a protecting roadway with a small coal pillar has been developed and applied for many years, and a satisfactory supporting effect has been obtained in medium–thick coal seam and thin coal seam mining. However, the gob-side roadway or small coal pillar mining in a thick coal seam is still subjected to technical problems occasioned by factors such as high roadway, high support pressure beside roadway, and waste of coal resources. To solve these problems, the author proposes an innovative technology of coal-free mining: the technology of driving roadway along goaf with a flexible formwork pre-cast wall. The article utilizes the 3503 and 3505 working faces of Wangzhuang Coal Industry Group as the research background, and comprehensively introduces the principle of the technology and the overburden rock movement law. Through theoretical calculations and numerical simulations, the support resistance and support parameters of flexible formwork pre-cast walls have been determined and successfully performed in industrial practice. The results indicate that the combination of the flexible mould pre-cast wall coal pillar-free mining technology and roof cutting process is more conducive to the maintenance of the roadway in the lower working face, and effectively reduces the stress and deformation of the surrounding rock. The roof and floor of the drivage roadway move, and the deformation of the two sides is small; furthermore, the overall roadway retention effect is satisfactory, which meets the requirements of mining in the lower working face. The coal pillar pertaining to the 20 m section of the 5 m high mining height face was recovered for Wangzhuang Coal Mine, and the recovery rate of the coal resources and the driving speed of the roadway were improved. The proposed method can be popularised and applied in this mine and even in the mining of 15# large-height coal seams in the two cities.

## Introduction

China is a large consumer of coal resources, and at this stage, the production of major minerals is still growing. Total primary energy production in the past 2022 was 4.43 billion tonnes of standard coal, with coal accounting for 67.0% of the energy production mix. The increased demand for coal resources has promoted the rapid development of coal mining technology^1^, and owing to the continuous improvement of the mechanisation level, the production efficiency of coal mines has also been immensely improved^2^. However, in general, when mining large high coal seams, it is necessary to leave coal pillars between the comprehensive mining face to protect the safety of the face mining^3,4^, which also occasions a certain waste of coal resources. How to safely and efficiently recover the coal pillars of the large mining face, or no longer leave coal pillars without affecting the succession of the face and still be able to mine safely, has become an urgent mining problem^5^. To solve the aforementioned problems, researchers have proposed the method of mining without coal pillars or small coal pillar mining^6,7^.

**Figure 1:**
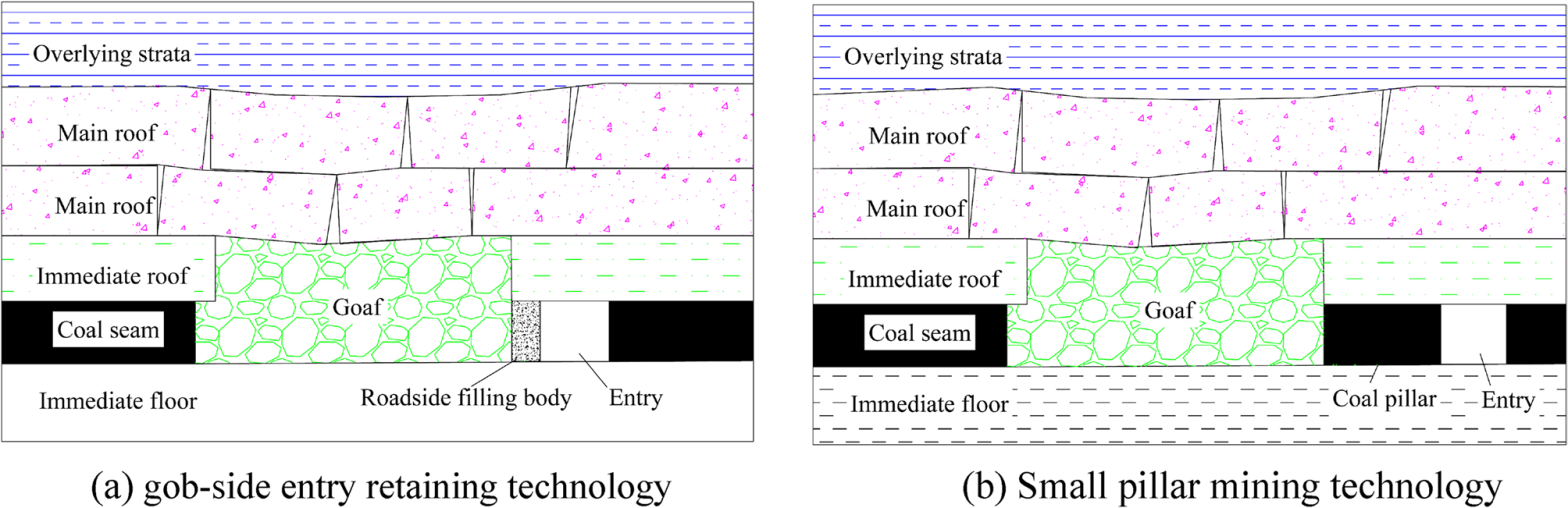
Technical profile of mining. (**a**) Gob-side entry retaining technology, (**b**) small pillar mining technology.

Currently, the more widely utilized coal pillarless mining technology is gob-side entry retaining technology, herein depicted in Fig. 1a. The technology fundamentally entails maintaining the post-mining roadway behind the working face along the boundaries of the void zone; in most scenarios, to ensure that the effectiveness of the post-retention roadway, engineers construct another filling wall along the side of the void zone of the retention roadway^8–15^. This method improves the coal resource recovery rate on one hand, and on the other hand, it also realizes Y-type ventilation and effectively manages the gas overlimit problem in the working face^16–18^. However, gob-side entry retaining technology cannot be completely applicable to all working conditions; when the coal mining depth increases, or the working face mining height is large, phenomena such as crucial deformation of the roadway or gob-side entry retaining wall cracking occurs, which critically undermines roadway construction safety^19^. The aforementioned observation can be rationalized as follows: along the air retaining alley wall that offers passive support, wall early strength is generally low; when the roof plate transient pressure exceeds the wall initial support capacity, the surrounding rock deformation becomes more critical, and the effect of the final roadway retention is increased^20^.

Leaving small coal pillar mining technology is also one of the current methods for improving the recovery rate of large-height coal resources and solving the problem of succession tension, as depicted in Fig. 1b. This technology is essentially based on the distribution law that regulates the lateral support pressure of the return mining face, the scientific arrangement of the return mining roadway, and the arrangement of the return mining roadway within the influence of the internal stress field^21–24^. This not only minimizes the size of the coal pillar in the section, but also improves the extraction rate of coal resources from thick or extra thick coal seams. However, small pillar mining is still accompanied by the waste of coal resources, especially in the large mining height working face, which often leads to high level coal-resources loss. When the mine belongs to categories such as the water-rich roof, gas protruding mine, and easy to spontaneous combustion coal seam, the mining method of leaving small coal pillars becomes even more inapplicable.

In regard to the deficiency of non-coal pillar mining or the small coal pillar mining technology of retaining roadway along goaf, this study proposes an innovative non-coal pillar mining technology: driving the roadway along goaf with a flexible formwork pre-cast wall. This technology is different from the method of retaining the filling body along the goaf. The location of its filling body is chosen to be close to the side of the solid coal of the adjacent working face, which exhibits a high bearing capacity; therefore, the flexible formwork wall left in the protection pertaining to the solid coal of the adjacent working face does not require a high bearing performance in the early stage. To be adjacent to the working face back to the mining roadway excavation period, leave the wall has a high bearing capacity: the maintenance of the roadway after the excavation and the disaster management of the hollow area is also more favourable. The advantages and disadvantages of this technology compared with gob-side roadway mining without coal pillar mining and small coal pillar mining are depicted in Table 1.

**Table 1:** Comparison of advantages and disadvantages of mining technologies.

Mining technology	Advantages	Disadvantage
Gob-side entry retaining	1 roadway less excavated	Experienced 2 mining impacts
	Recovery section coal pillar	The supporting body is easily damaged by pressure in the early stage
	Close goaf and effectively control gas	Poor effect of retaining roadway with high mining height or deep burial depth
Driving roadway with small coal pillar	Improve coal resource recovery rate Reduce support costs	Waste of coal resources
		Not conducive to goaf management control
		Excavate 1 more roadway
Flexible formwork pre-cast wall driving roadway along goaf	Recovery section coal pillar	Widen the roadway in advance Excavate 1 more roadway
	Close goaf and effectively control gas	
	The effect of roadway formation is satisfactory with large burial depth or high mining height	
	High strength of the support body	

This study utilizes Wangzhuang Coal Industry (5 m large mining height; 3503 working face) as the research background. It systematically introduces the innovative non-pillar mining technology principle, the movement characteristics of overlying rock, as well as the technical design scheme and engineering application of roadway driving along goaf with a flexible formwork pre-cast wall. This research aims to solve the technical problems occasioned by the high roadway and high pressure of roadside support in the large-height working face of thick coal seam along the hollow stay roadway and can provide a novel technical choice for how to recover the coal pillar in the 20 m section of the 5 m high mining face, thereby improving the recovery rate of coal resources and the roadway’s driving speed. Thus, the study promotes the application in this mine and even in the mining of the 15# coal seam in the two cities.

## The principle of the technology of driving roadway along goaf with a flexible formwork pre-cast wall

### Flexible formwork closed wall structure

#### Flexible formwork for textile structures

**Figure 2:**
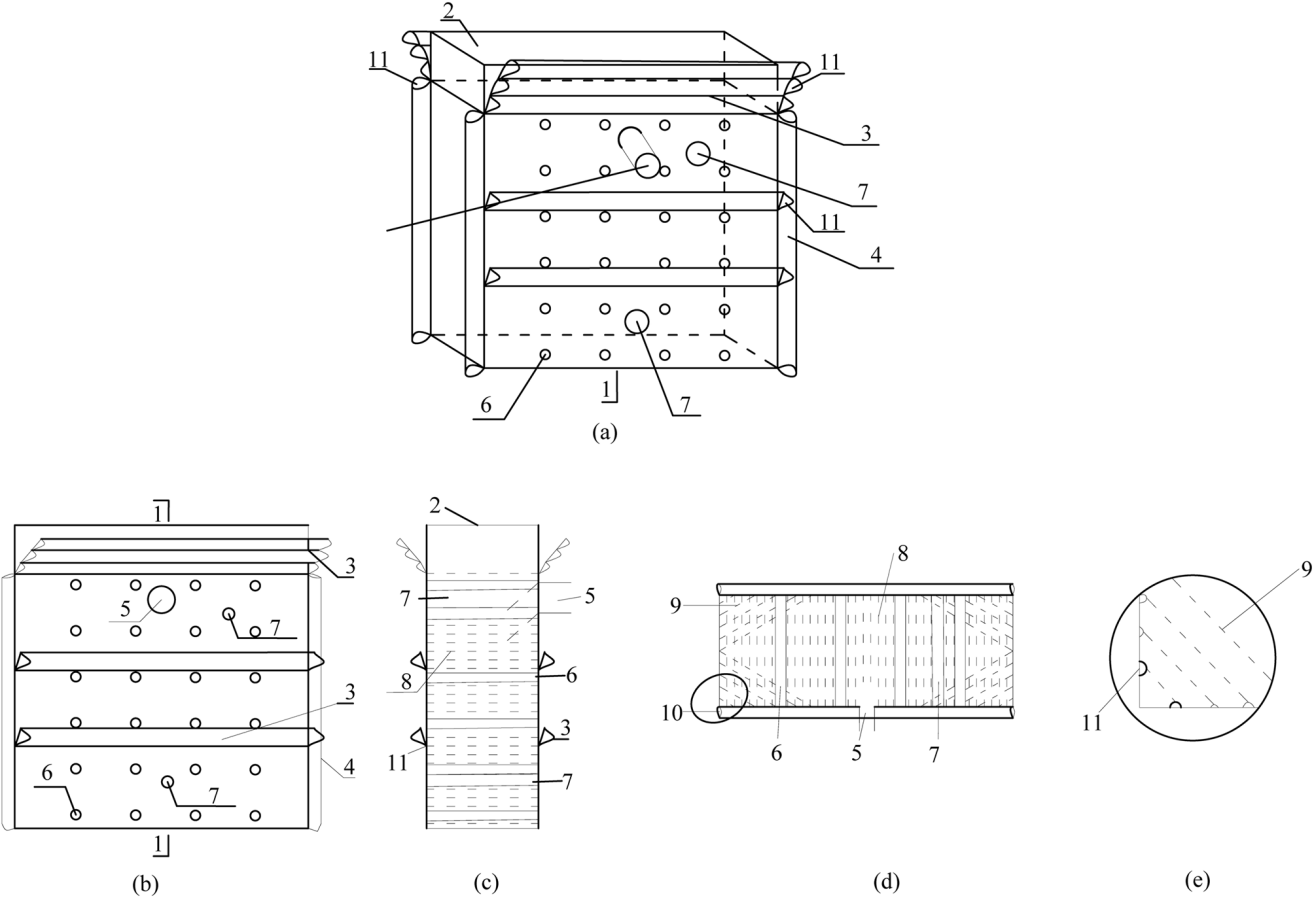
Flexible formwork for textile structures: (**a**) three-dimensional view, (**b**) main view, (**c**) left view, (**d**) top view, and (**e**) partial diagonal tension bar. 1—Body formwork, 2—jointed top formwork, 3—transverse flange, 4—vertical flange, 5—grouting opening, 6—anchor bolt holes, 7—preparation holes, 8—transverse tension reinforcement, 9—diagonal tension reinforcement, 10—sleeve, 11—reinforcement ring.

Flexible formwork for textile structures is referred to as flexible formwork, as shown in Fig. 2. Which exhibits the following characteristics:

- Flexible concrete is a typical concrete formwork that can be formed by itself, exhibits characteristics such as one-time use without recycling and simple operation of supporting and hanging, and can form a combined structure that entails a flexible concrete confined wall merged with concrete, anchors, and a reinforcing steel mesh.
- The flexible formwork is internally equipped with transverse and longitudinal tie bars and anchor bolt holes to optimize the wall’s stress distribution, increase the modulus of concrete, alter its strength and deformation state, limit lateral displacement, and increase the frictional resistance between concrete and other materials, thereby improving the stability of the concrete and the flexible formwork closed wall support structure.
- The flexible formwork design, which can set up gas drainage holes, drainage holes, and observation holes according to technical requirements, consists of two components, namely the main formwork and the top formwork, which are forcibly connected by pump pressure to isolate the goaf. The combined structure of the flexible-formwork closed wall after being connected to the top achieves high water to cement ratio transportation and rapid solidification at a low water to cement ratio due to its permeable and non-permeable properties.
- The flexible formwork confined wall assemblage structure after jointing the roof achieves a large water ratio delivery and a small water-cement ratio for rapid curing owing to its permeable and impermeable slurry properties.

#### Flexible formwork pumping concrete

Flexible formwork pumped concrete consists of textile structural flexible formwork and specialised pumped concrete. The special pumped concrete is a kind of self-compacting concrete, which is mainly composed of medium sand, crushed stone, cement, fly ash, water, and a special admixture for flexible moulded concrete, and the proportion is depicted in Table 2.

**Table 2:** Proportioning of specialised pumped concrete.

Materials	Cement	Sand	Gravel	Fly ash	Water	Admixture
Mass (kg)	400	700	700	50	200	0.8

**Figure 3:**
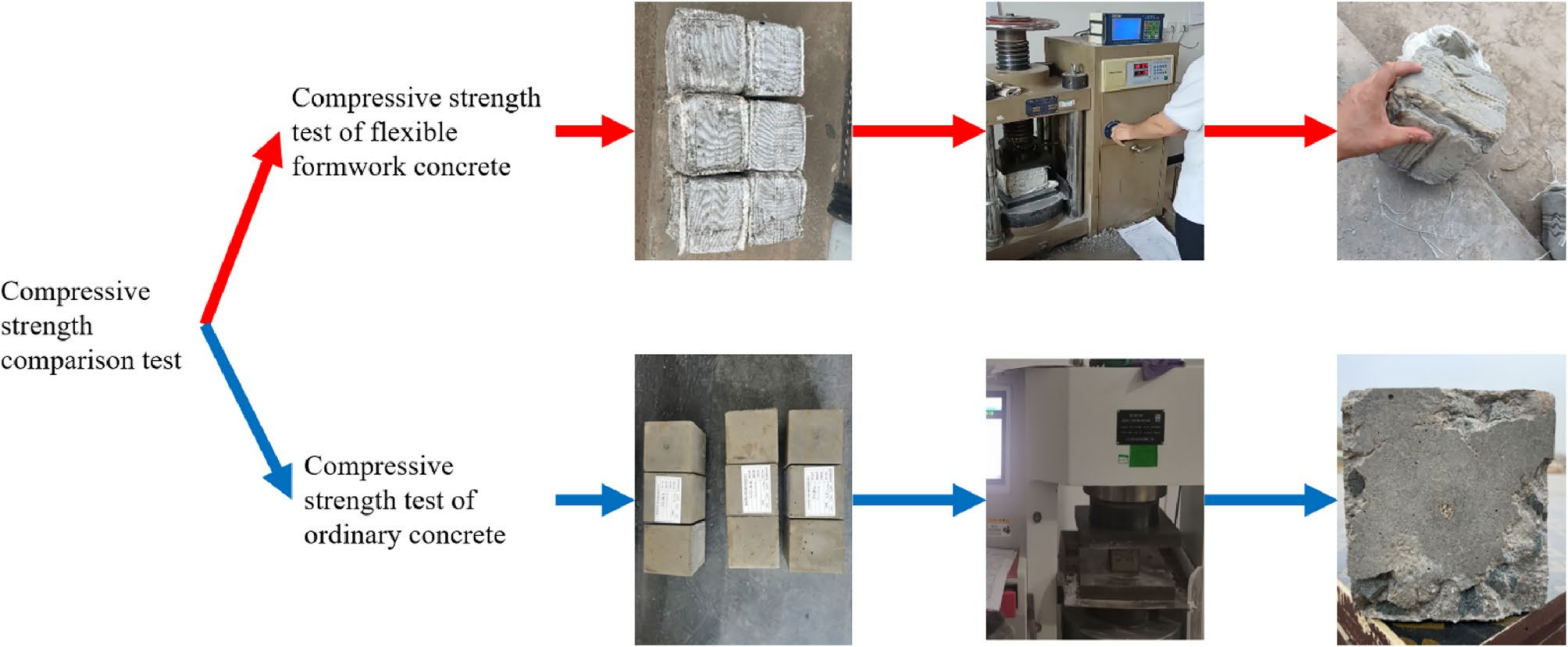
Comparison test of uniaxial compressive strength.

**Figure 4:**
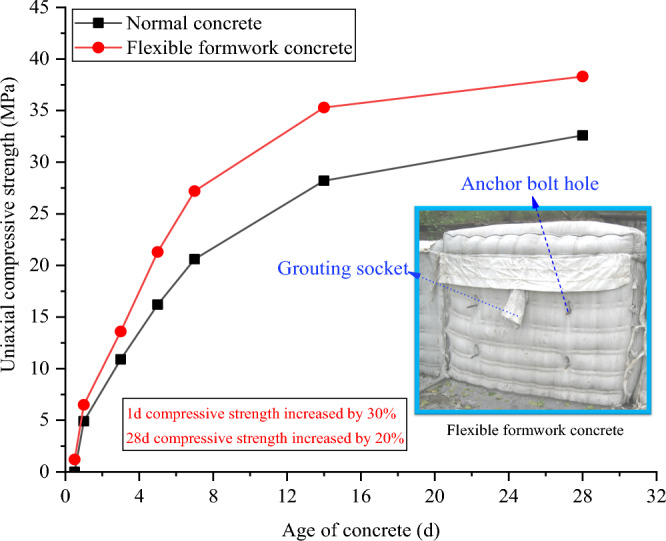
Relationship curve between concrete age and compressive strength.

The continuous closed wall formed by flexible formwork pumping concrete is mainly utilized to support the roof in a timely manner, close the goaf, preserve the roadway, and serve the next working face. Thus, the flexible formwork pumping concrete wall must exhibit a high initial support force. Through the laboratory concrete mechanical properties test research, it was found that the flexible formwork pumped concrete 0.5 day uniaxial compressive strength was 0.8 MPa, 1 day uniaxial compressive strength relative to ordinary concrete increased by 30%, and 28 days uniaxial compressive strength increased by 20%. The uniaxial compressive strength test procedure is illustrated in Fig. 3. A control plot of the curve obtained from the tests for the change in compressive strength of concrete with age is illustrated in Fig. 4.

### Technology of driving roadway along goaf with flexible mold pre-cast wall

**Figure 5:**
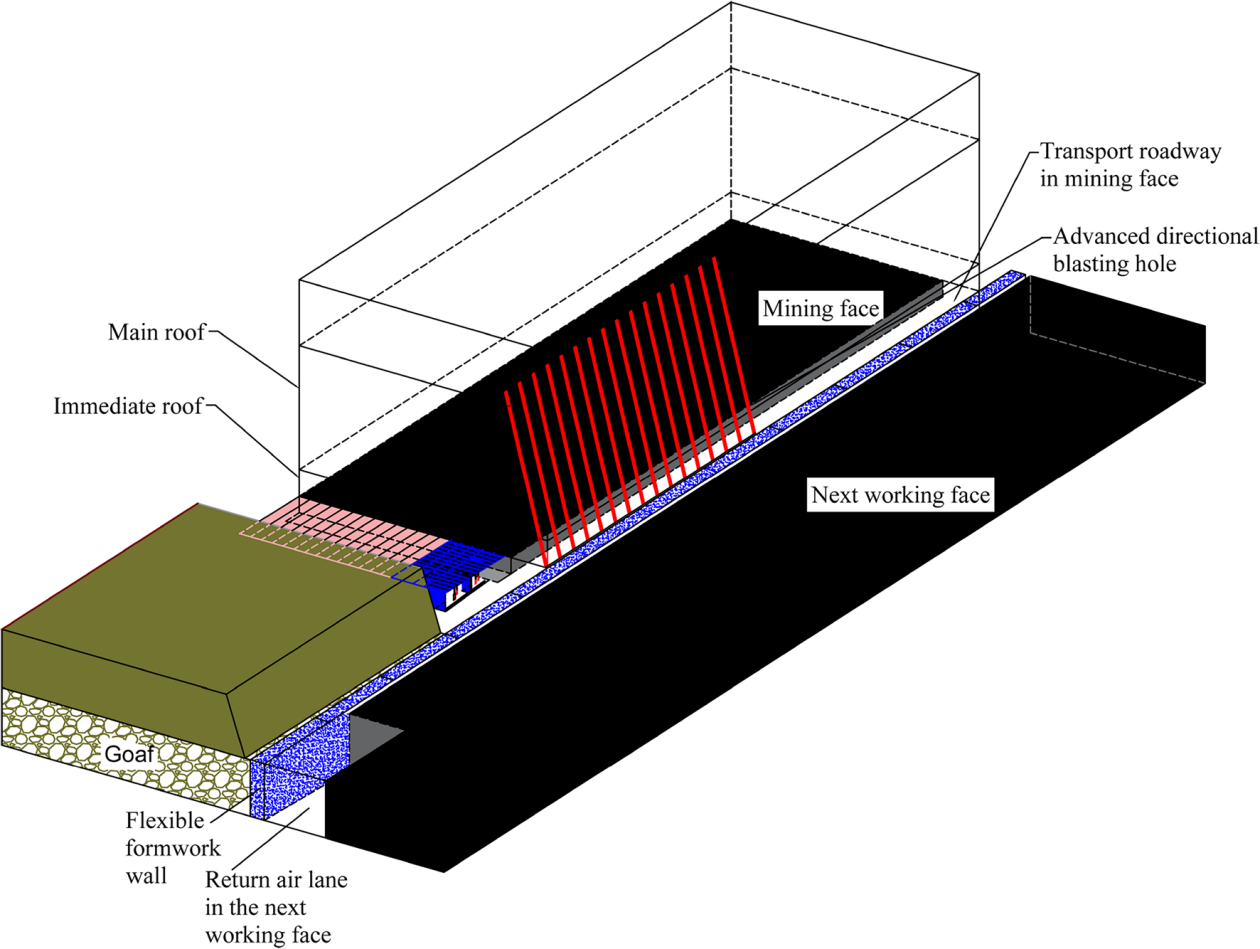
3D diagram of retaining wall along goaf. (**a**) Roadway before mining in the last working face, (**b**) expanding roadway, (**c**) implement flexible formwork pre-cast wall technology, (**d**) before driving the roadway in the next working face, (**e**) cutting top pressure relief process, (**f**) after driving the roadway in the next working face.

**Figure 6:**
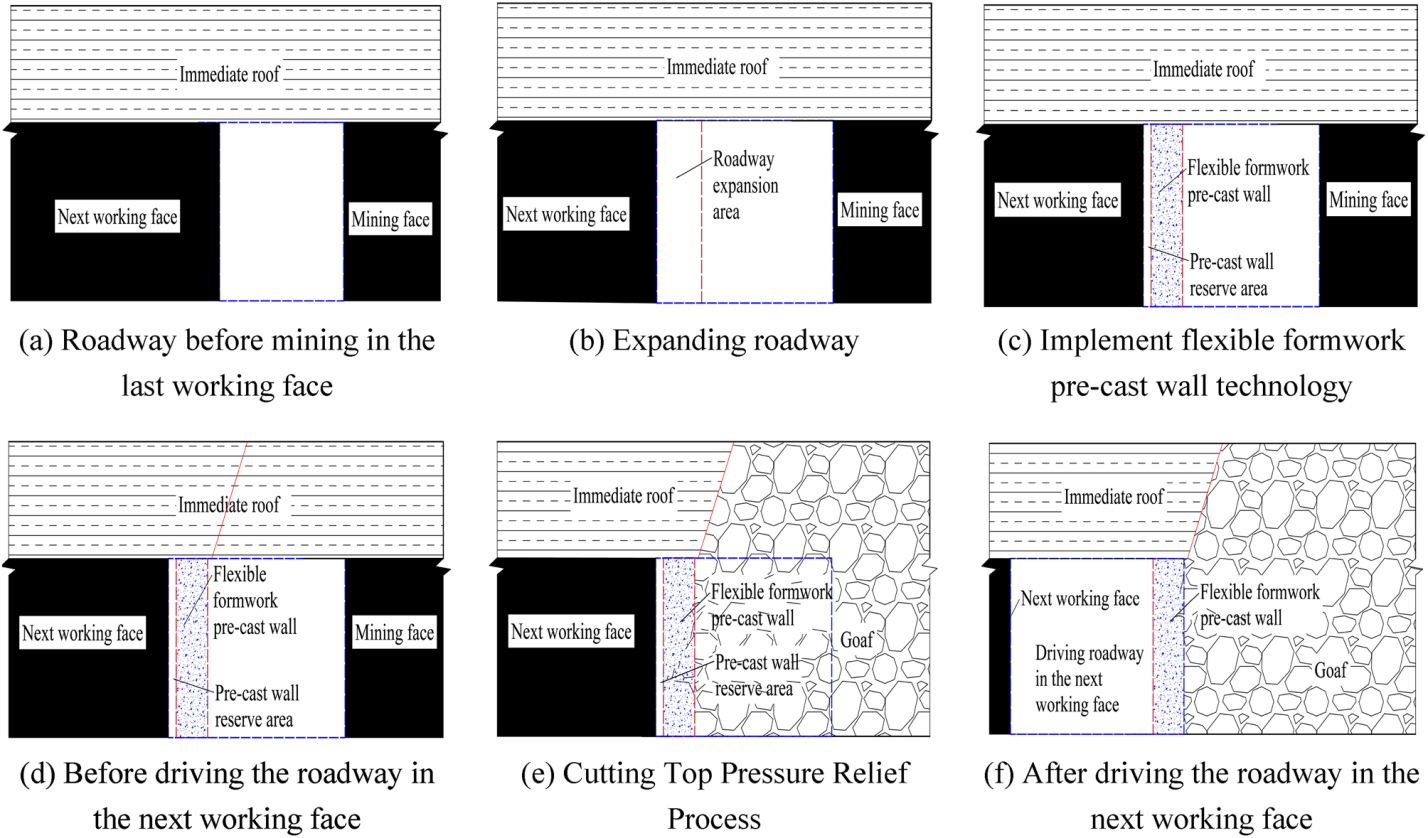
Process profile of retaining wall along goaf.

According to the construction process, the technology of driving roadway along goaf with a flexible formwork pre-cast wall can be categorized as follows: pre-cast wall and driving roadway along the wall. This technology is mainly applied to non-pillar mining in the mining face with large mining height or complex conditions. The main principle is explained as follows: Before the face is mined back, the outer gang side of the transport road is expanded in advance at a certain distance (not less than 50 m) in front of the face, and a continuous flexible formwork closed wall structure is subsequently poured in the expanded area. After the previous face is mined back (not less than 300 m), and when the rock layer activity of the air-mining area tends to be stabilised, the next face is dug in the return road immediately adjacent to the continuous flexible formwork closed wall, thus realizing the mining without coal pillars along the goaf retaining wall. Because the wall has to be affected by three times of mining, if the wall is critically damaged, to reduce the wall pressure after mining and ensure the integrity of the wall, presplitting blasting in advance should be adopted to relieve pressure, shorten the working face cycle to press the step distance and the length of lateral cantilever beam, reduce mining pressure, reduce the deformation and destruction of roadway along goaf, and ensure that the roadway along goaf can meet the production requirements of the working face. The construction technology of retaining wall along goaf is depicted in Fig. 5, and the section drawing is depicted in Fig. 6.

### Movement characteristics of overlying rock and mechanical structure model of surrounding rock in roadway driving along goaf with a flexible formwork pre-cast wall

#### Analysis on the movement characteristics of overlying rock in roadway driving along goaf with a flexible formwork pre-cast wall

Driving roadway along goaf with a flexible formwork pre-cast wall is a dynamic process, which can be divided into three stages^25–27^, namely early stage, transition period, and late stage, according to time. With the advance of the mining face, there is a disturbance in the early stage, and the wall side roof is deformed by rotation and subsidence owing to the direct roof collapse and the basic roof bending and subsidence. During the transition period, the roof of the wall left along the goaf is still dominated by the rotational deformation of the direct roof and the basic roof, which is characterized by high speed and large deformation. In the later stage, the roof deformation is mainly sinking, exhibiting a certain volatility, which is characterized by long duration and low sinking speed. Therefore, to ensure that the wall has a higher bearing capacity and the integrity of the wall will not be destroyed in the early stage and transition period, according to the strength growth change of flexible formwork concrete, the wall pouring should be no less than 50 m in advance of the mining face.

**Figure 7:**
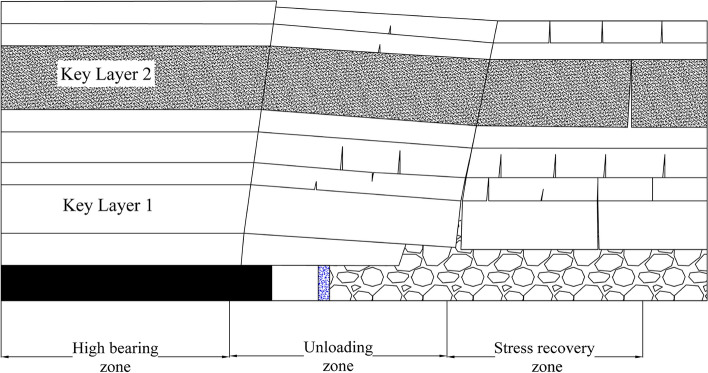
Distribution of the lateral stress area in the stope.

The roadway driving along the wall must be performed when the advance of the mining face is not less than 300 m. Meanwhile, the collapse of the roof strata in the pre-pouring wall stage has gradually stabilized, generally attaining the height of the caving zone and fracture zone, and the overlying strata load is transferred to the lower strata through the lateral roof, leading to stress concentration in a certain range of the coal body. Under the protection of the masonry beam structure in the driving roadway area, the surrounding rock stress has been somewhat released, and the stress curve is in low stress area. The gangue in the goaf is further compacted under the overlying load, and the stress is gradually restored. Consequently, as depicted in Fig. 7, the quarry creates three stress zones in the lateral direction in sequence: the high bearing zone, the unloading zone, and the stress recovery zone.

#### Mechanical model of the surrounding rock structure of roadway driving along goaf with a flexible formwork pre-cast wall

The principle of flexible formwork pre-cast wall driving roadway along goaf indicates that the flexible formwork concrete confined continuous wall is poured close to the coal wall of the lower face; after mining in the upper face, the wall is affected by the first mining, and when the mining area is subsequently stabilised, it will be close to the continuous flexible mould confined wall tunnelling into the return airway of the next face, and, meanwhile, the wall is affected by the second adoption, which ultimately forms a complete tunnel that can be utilized in the next face back to the mining service. Therefore, the establishment of the structural mechanical model of surrounding rock should be based on the period after the stability of the driving roadway in the following working face.

**Figure 8:**
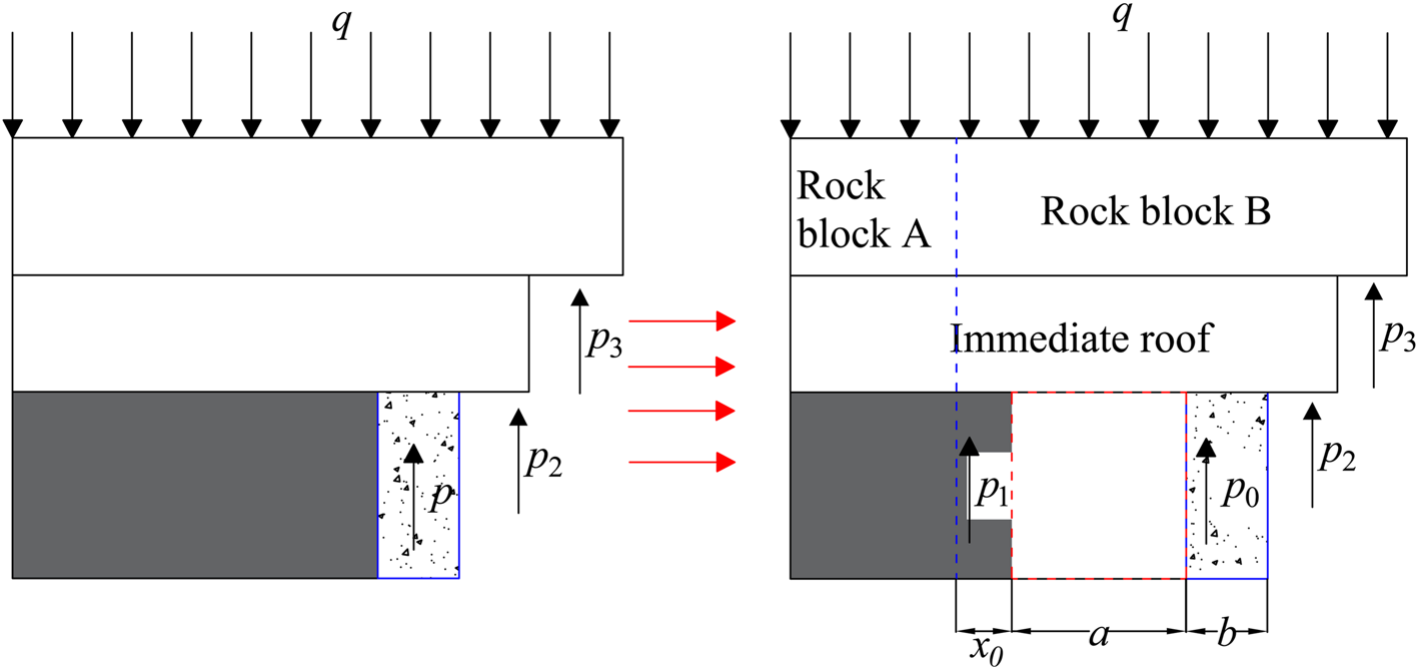
Mechanical model of roadway driving along goaf with a flexible formwork pre-cast wall.

The following assumptions are made based on the movement characteristics of the overburden: the basic roof takes the elastic–plastic junction of solid coal as the rotation axis and is inclined to the goaf. Shear stresses are negligible at the direct roof, basic roof, and with the more superior rock layers; the gangue fills the mining area and acts as a support for the direct and basic roof rock formations; the weight of the weak rock layer above the basic roof is uniformly applied over the basic top; the roof pressure is uniformly applied to the roadside support body; and the effect of in-roadway support is neglected (flexible formwork concrete roadside support is much greater than in-roadway support)^28^. Therefore, a mechanical model can be built as illustrated in Fig. 8, where *q* denotes the basic top rock action force; *p*_0_ denotes the lane-side support resistance; *p*_1_ denotes the support resistance pertaining to the coal body of the lane gang to the roof; *p*_2_ denotes the support resistance of collapsed gangue to the direct roof; *p*_3_ denotes the support resistance of collapsed gangue to the basic roof; *x*_0_ denotes the width of the solid coal limit equilibrium zone of the lane gang; *a* denotes the width of driving roadway; and *b* denotes the width of the supporting body beside the roadway.

Figure 8 indicates that the wall and coal side before roadway excavation are subjected to the load of the overlying strata and the supporting resistance of the wall and coal side, as well as the supporting force of gangue in the goaf, thereby forming a mechanical equilibrium. The formation of a new roadway after driving exerts a disturbing effect on the mechanical equilibrium, based on which the mechanical equilibrium equation in the vertical direction after driving the roadway can be established:

1 $$ p_{1} x_{0} + p_{0} b + p_{2} l + p_{3} l_{1} + = \gamma_{1} m_{1} L_{1} + \gamma_{2} m_{2} L_{2} + qL_{3} $$

where *l* denotes the length of the collapsed gangue on the direct roof; *l*_1_ denotes the length of the collapsing gangue acting on the basic roof; *γ*_1_ denotes the capacitive weight of the basic roof; *γ*_2_ denotes the capacitive weight of the direct roof; *m*_1_ denotes the thickness of the basic roof; *m*_2_ denotes the thickness of the direct roof; *L*_1_ denotes the length of rock mass B; *L*_2_ denotes the overhang length of the direct roof; and *L*_3_ denotes the length over which the uniform load acts.

Because* p*_1_ is equal to the residual compressive strength *σ*_c_ of the coal body in the loose area of the roadside gang, considering the stress concentration factor *K*; meanwhile, the support resistance *p*_0_ of the roadside support body is

2 $$ p_{0} = K\left[ {\gamma_{1} m_{1} L_{1} + \gamma_{2} m_{2} L_{2} + qL_{3} - \sigma_{c} x_{0} - p_{2} l - p_{3} l_{1} } \right]/b $$

The expression for × 0 in the equation is^29^:

3 $$ x_{0} = \frac{\eta m}{{2\tan \varphi }}\ln \left[ {\frac{{K\gamma ^{\prime}H + \frac{c}{\tan \varphi }}}{{\frac{c}{\tan \varphi } + \frac{{\sigma_{c} }}{\eta }}}} \right] $$

where *η* denotes the lateral pressure coefficient; *m* denotes the thickness of the coal seam; *γ*' denotes the average bulk weight of the overlying rock formation; *φ* denotes the angle of internal friction at the interface between the coal seam and the top and bottom slab; *c* denotes the cohesive force at the interface between the coal seam and the top and bottom slab; and *H* denotes the burial depth of the coal seam being mined.

According to the elastic foundation theory, the support resistance of the collapsed gangue in the goaf to the direct roof and the basic roof is

4 $$ p_{2} = \frac{{\gamma_{2} m_{2} }}{2} $$

5 $$ p_{3} = \frac{{\gamma_{1} m_{1} }}{2} $$

#### Calculating the bearing capacity of flexible formwork walls

The flexible formwork concrete wall is a fibre wrapped reinforced pre-stressed composite structural body. Therefore, the bearing capacity of the flexible formwork concrete consists of two components: the restrained reinforcement and the core concrete.

Under the action of axial pressure, the restrained core concrete produces transverse expansion deformation; thus, the anchor bolt produces tensile deformation, thereby forming a transverse binding force acting on the core concrete, which is in a three-way compressive stress state. At the beginning of loading, the stress–strain relationship of flexible formwork concrete is basically the same as the curve of ordinary plain concrete; once the stresses have attained the concrete’s strength, the curve of the flexible formwork concrete maintains a certain upward trend; eventually, the curve ends with a more gentle downward leg.

Due to this considerable lateral expansion deformation occurring only after the axial compressive strength of the concrete is attained, the stress–strain relationship curve of flexible formwork concrete exhibits significant differences only when the stress exceeds the compressive strength of the concrete, thereby forming a strengthening effect. The strength and ductility of flexible formwork concrete is improved compared to plain concrete. Therefore, the bearing capacity of the flexible formwork concrete wall is calculated as

6 $$ N = 0.9\varphi (f_{c} + 4\sigma_{r} )A_{c}{\prime} $$

where *σ*_*r*_ is expressed as follows:

7 $$ \sigma_{r} = \frac{{\pi d^{2} \cdot \sigma_{b} }}{{4a_{1} \cdot a_{2} }} $$

where *N* denotes the bearing capacity of the flexible formwork wall; *φ* denotes the stability factor of the member; *f*_*c*_ denotes the design value of concrete axial strength MPa; *σ*_*r*_ denotes the effective binding force produced by the action of the anchor bolt ferrule, MPa; *A*_*c*_' denotes the cross sectional area, m^2^; *d* denotes the diameter of the anchor bolt, mm; *σ*_*b*_ denotes the design value of anchor bolt tensile strength, MPa; and *a*_1_ and *a*_2_ denote the spacing and row spacing of the anchor bolts, respectively, mm.

## Engineering overview

### Engineering background

**Figure 9:**
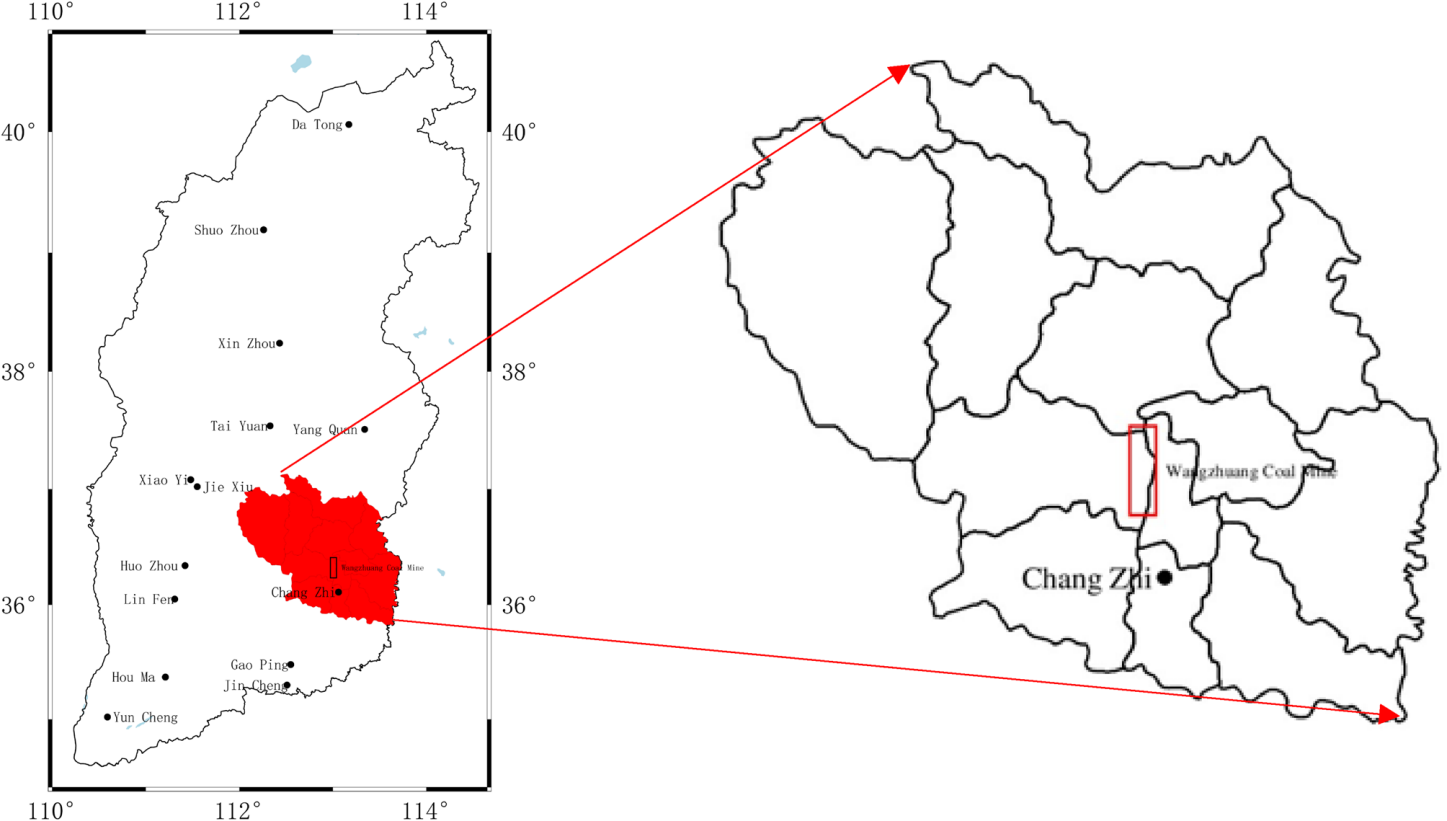
Location map of Wangzhuang coal mine.

Shanxi Changzhi Wangzhuang Coal Industry Limited Liability Company (referred to as Wangzhuang Coal Mine), which is located 26 km south of Changzhi City and 13 km southeast of Shangdang District in Shanxi Province, China, is subordinate to Shanxi Coal Transportation and Marketing Group Co Ltd, as illustrated in Fig. 9. Wangzhuang Coal Mine is approved to mine the 3# and 15# coal seams, and currently mainly mines the 3# coal seam with a 2.4 million t/a production scale. The thickness of the No. 3 coal seam is 4.2–5.5 m (average thickness of 5 m); the dip angle of the coal seam is 2°–4°, which is near horizontal coal seam; and the mining method is large mining height and one time mining full height. Currently, there are only two coal mining workings with large reserves left in the No. 3 seam, namely 3503 and 3505, which are immediately adjacent to the No.3 seam. The workings are left with 20 m of coal pillars in the section, and the recoverable reserves are less than 8 million tonnes, with a service life of only approximately 3 years left. After the depletion of 3# coal resources, the mine switches to mining 15# high sulphur coal. In regard to the mines in Changzhi City and Jincheng City in Shanxi Province that are currently mining the 15# coal seam, almost all of them are undergoing financial loss. The recovery of the coal pillar pertaining to the 20 m section of the 5 m high working face and the improvement of the recovery rate of coal resources and roadway boring speed have become major engineering and technical problems faced by Wangzhuang Coal Mine. In the scenario where the highly flexible formwork concrete wall along the goaf is not suitable for large-scale mining, it is urgently necessary to test new pillar free mining technology in the currently more cost-effective 3 # coal 3503 and 3505 working faces. Along these lines, in this work, a flexible mold pre-cast wall driving roadway along the goaf without coal pillar mining technology was independently developed. This method is expected to be popularized and applied in the mining of coal seam in Wangzhuang Coal Mine, and even in two cities.

### Overview of the working face

**Figure 10:**
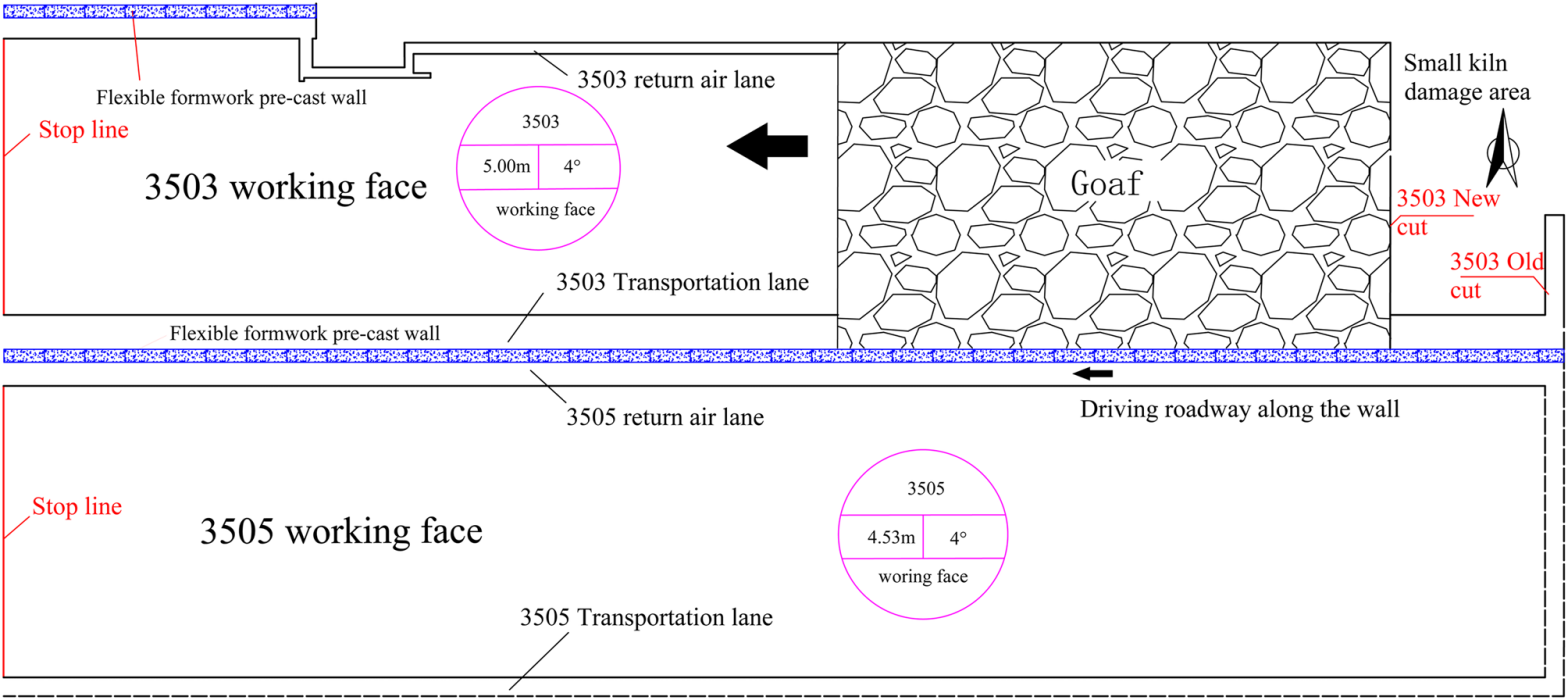
Working face roadway layout.

The 3503 working face belongs to the 35 mining area, the surface of which is the Old Xiongshan Mountain, and there are no buildings or other facilities on the ground. The working face is located at a level of + 980, with an elevation of + 899.4  to  + 970.5 m, an advancing length of 1265 m, and a dip length of 307.75 m. It is also adjacent to the 3502 goaf to the north, a small kiln damage area (geophysical exploration results indicate a rich water area) to the east, an unexplored 3505 working face (without excavation roadway) to the south, and a rubber wheel roadway in the 35 mining area to the west. The roadway with retaining walls along the goaf is the transportation roadway of the 3503 working face, and the specific layout of the working face is illustrated in Fig. 10.

**Figure 11:**
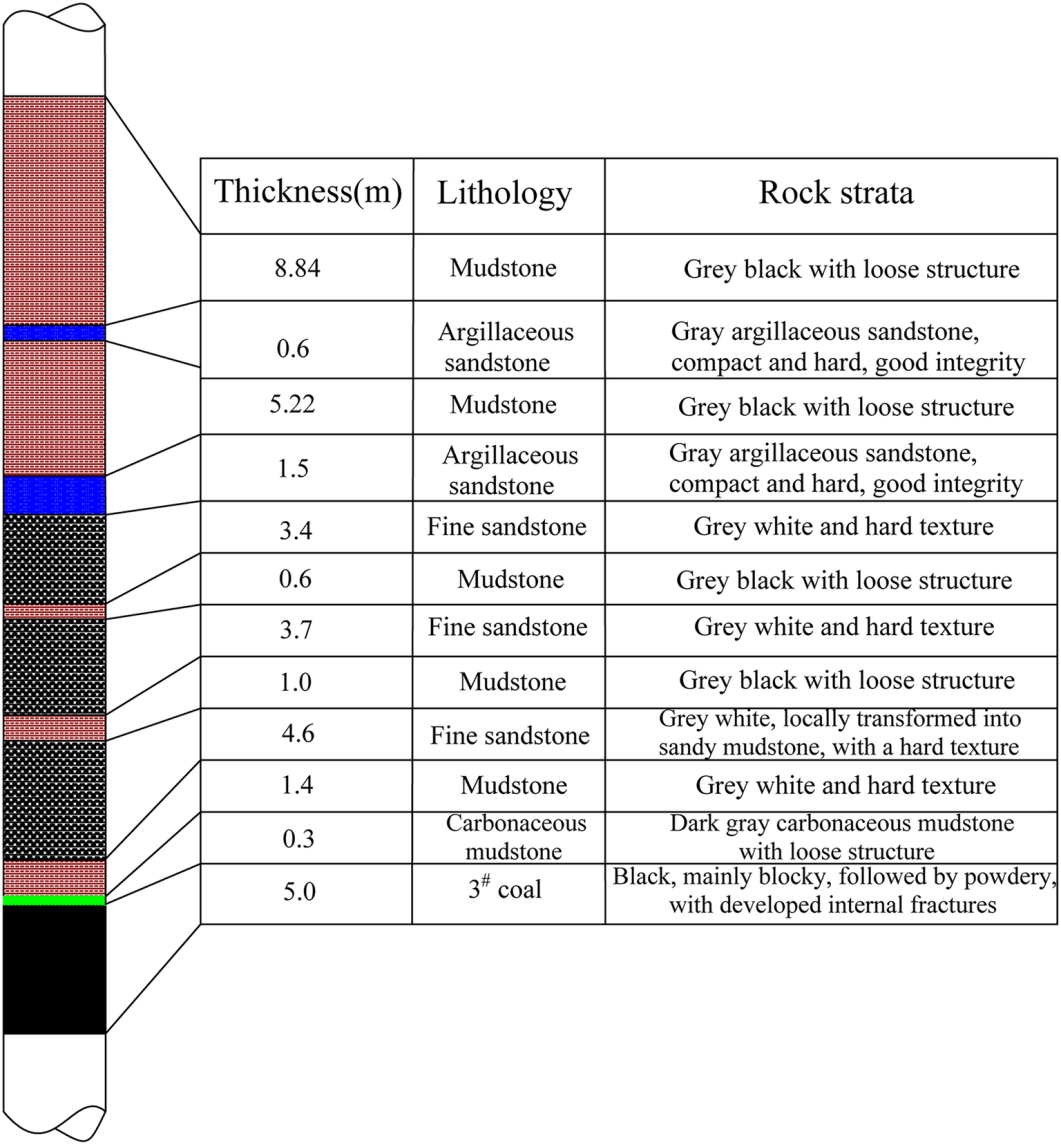
Columnar diagram of the roof strata.

The 3503 transport road tunnelling coal seam for the 3 # coal, commonly known as "fragrant coal", is endowed with the lower Permian Shanxi Group strata, the coal seam to the main block, powder to the second, strip marks for the black, jagged—step fracture, and endogenous fissure development. The thickness of the coal seam is 4.20–5.50 m (average thickness of 5 m), and its dip angle is 2°–4°. The coal seam contains 0–3 layers of interbedded gangue: the interbedded gangue is composed of mudstone. The thickness of gangue is 0.27–0.3 m, and the columnar diagram of the rock layer on the top of the coal seam is depicted in Fig. 11.

## Numerical simulation of roadway driving along goaf with flexible formwork pre-cast wall

### Calculation of parameters of roadway driving along goaf with a flexible formwork pre-cast wall

- Calculation of rock loads in each stratum of the lateral roof plate
  To obtain the cantilever length of the lateral rock strata in the goaf, the load of each layered rock layer is required first. Since the thickness and lithology of each stratum varies, the load on stratum i can be determined from Eq. (8):
  8 $$ (q_{n} )_{i} = \frac{{E_{i} h_{i}^{3} (\gamma_{i} h_{i} + \gamma_{i + 1} h_{i + 1} + \cdot \cdot \cdot + \gamma_{i + n} h_{i + n} )}}{{E_{i} h_{i}^{3} + E_{i} h_{i}^{3} + \cdot \cdot \cdot + E_{i + n} h_{i + n}^{3} }} $$
  where (*q*_*n*_)_*i*_ denotes the load applied to layer i by the n layers above layer* i*; *E*_*i*_···*E*_*i*+*n*_ denotes the modulus of elasticity of each stratification; *h*_*i*_···*h*_*i*+*n*_ denotes the layer thickness of each stratum; *γ*_*i*_···*γ*_*i*+*n*_ denotes the tolerance of each stratification; *i* = 1,2,3….. If it is concluded that (*q*_*i*+*n*+1_)_*i*_ < (*q*_*i*+*n*_)_*i*_, it is assumed that (*q*_*i*+*n*_)_*i*_ is the load applied to layer* i*, from which the length of the rock cantilever can be obtained.
  According to the physico-mechanical parameters of each formation, substituting into Eq. (8) yields the load of each stratified formation and the length of the cantilever beam, as illustrated in Table 3.

**Table 3:** Layered rock loads and length of cantilever beams.

Layers (top-down)	Name	Thickness	Rock load (kN)	Cantilever beam length (m)
11	Mudstone	8.84	350.78	23.732
10	Argillaceous sandstone	0.6	15.48	5.274
9	Mudstone	5.22	149.8	9.362
8	Argillaceous sandstone	1.5	39.6	8.769
7	Fine sandstone	3.4	133.12	15.270
6	Mudstone	0.6	15.2	2.98
5	Fine sandstone	3.7	113.8	15.109
4	Mudstone	1.0	25.7	4.159
3	Fine sandstone	4.6	203.3	14.084
2	Mudstone	1.4	36	4.991
1	Carbonaceous mudstone	0.3	7.58	1.722
  Table 3 Layered rock loads and length of cantilever beams.
  Based on the length and thickness of the roof cantilever beams, it can be concluded that the fracture form of each rock layer of the roof is as follows. The first layer of carbonaceous mudstone and the second layer of mudstone are gradually layered and fractured; the fourth layer of mudstone faults synchronously with the third layer of fine-grained sandstone; the mudstone of Layer 6 is faulted synchronously with the fine-grained sandstone of Layer 5. The 8th layer of argillaceous sandstone, the 9th layer of mudstone, and the 10th layer of argillaceous sandstone are synchronized with the fine-grained sandstone of Layer 7; and the 11th layer of mudstone broke off on its own.
- Calculation of resistance and load of roadside support
  Based on the site survey, the relevant parameters of Eqs. (1) to (7) can be obtained: *η* = 0.5, *m* = 5 m, *φ* = 32.4°, *k* = 2, *γ*' = 25.91kN/m^3^, *H* = 234 m, *c* = 2.2 MPa, *σ*_c_ = 0.53 MPa, *x*_0_ = 2.44 m, *l* = 5 m, *l*_2_ = 24 m, *γ*_1_ = 26.23 kN/m^3^, *γ*_2_ = 25.5 kN/m^3^, *m*_1_ = 13.3 m, *m*_2_ = 1.7 m, and b = 1.5 m. The calculated resistance of the flexible mould wall support is *p*_0_ = 20,638.9kN, and the design value of axial compressive strength of concrete should be not less than *f*_*c*_ = 14,252.1kN/m^2^. A specification check indicates that the axial compressive design value of C30 concrete is as follows: *f*_*c*_ = 14.3 MPa = 14,300 kN/m^2^. Therefore, it was determined that a C30 strength, 1.5 m width flexible moulded concrete wall was utilized to conduct the numerical simulation and industrial test studies.
- Calculation of cutting roof height
  Because the pre-cast wall experiences three times the mining dynamic pressure, to ensure the effect of leaving the wall, by leaving the roadway wall, the researchers also calculated the parameters of the cut roof protection, and the minimum cut roof height *H* can be calculated according to the following formula^30^:
  9 $$ H = \frac{M}{{K_{P} - 1}} $$
  where *H* denotes the minimum roof release height required to adequately fill the voided area; *M* denotes the mining height of the coal seam, take 5 m; and *K*_p_ denotes the crushing expansion coefficient of the top rock layer, take 1.25–1.35. After calculation, we can obtain *H* = 14.3–20 m; if we completely fill the hollow area, the roof collapse height is 14.3–20 m.
  Upon considering the actual conditions, it was finally decided that the height of the roof cut was 16 m, the angle of elevation was 65° (the angle between the hole and the roof plate, pointing to the direction of the working face's mining area), the length of the hole was 17.6 m, and the diameter of the hole was 75 mm.

### Establishment of the numerical model

#### (1) Model establishment

**Figure 12:**
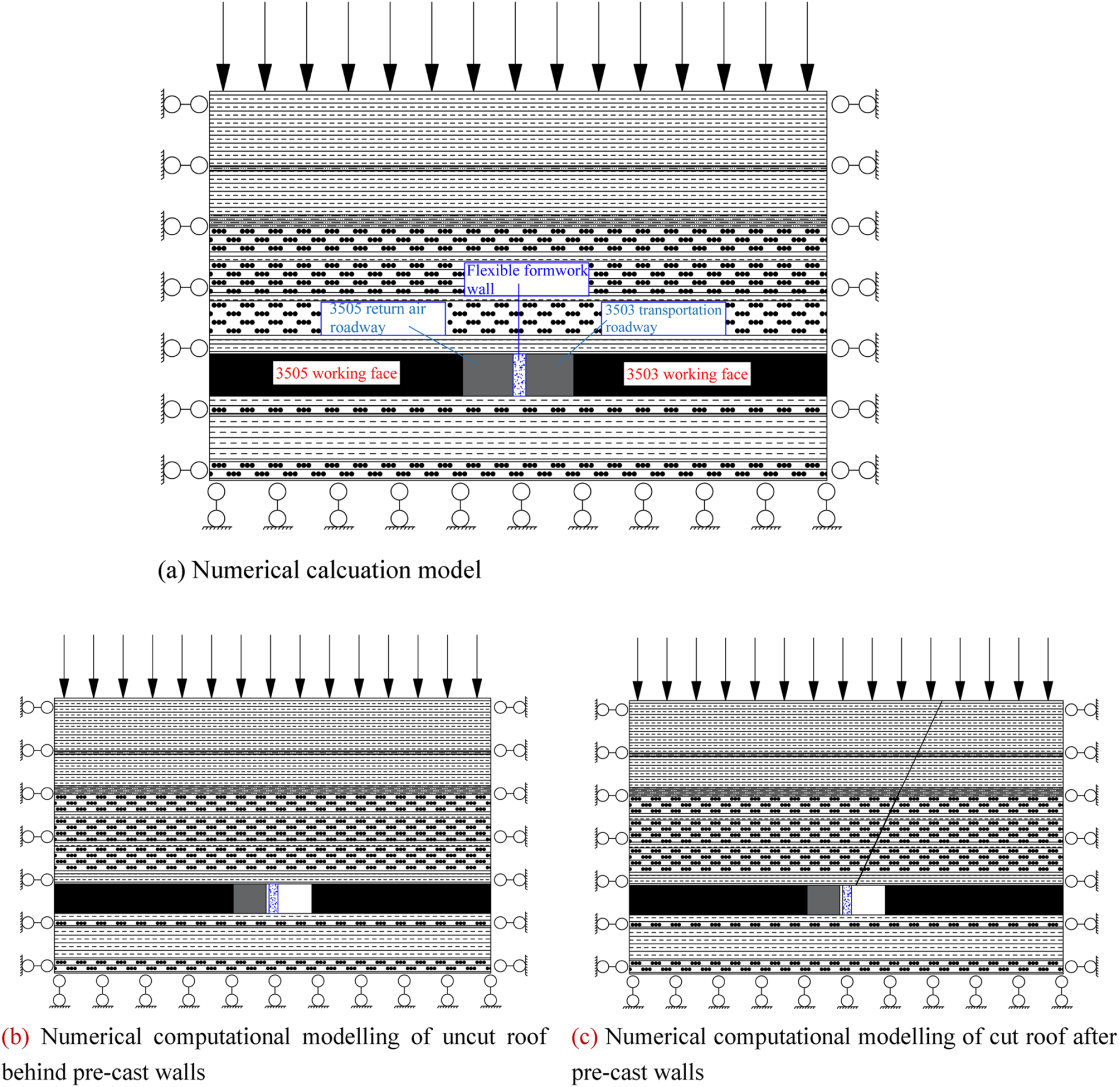
Numerical model. (**a**) Numerical calcuation model, (**b**) numerical computational modelling of uncut roof behind pre-cast walls, (**c**) numerical computational modelling of cut roof after pre-cast walls.

The working faces 3503 and 3505 of the Wangzhuang coal mine are utilized as a background. Based on the theoretical analysis and numerical calculation results, Flac3D numerical simulation software was utilized to analyse the stress distribution law of the flexible formwork concrete wall in the 3503 working face after mining back under the condition of no roof cutting and roof cutting. The stress and deformation law affects the surrounding rock of the roadway under the condition of no roof cutting and roof cutting after driving the roadway in the 3505 working face. Through the analysis and comparison of numerical simulation, the support effects of non-roof-cutting and roof-cutting are compared, which provides support for the design and industrial test of coal-free mining of roadway driving along goaf with a flexible formwork pre-cast wall. According to the geological conditions of the 3503 working face in Wangzhuang coal mine (see Table 4), a numerical simulation model was established as illustrated in Fig. 12.

**Table 4:** Geological conditions of the 3503 working face in Wangzhuang coal mine.

Lithology	Thickness (m)	Elastic modulus (GPa)	Poisson's ratio	Bulk density (kN/m^3^)	Cohesive force (MPa)	Internal friction angle (°)	Tensile strength (MPa)
Mudstone	8.84	14.3	0.2	25.73	7.2	24.4	4.21
Argillaceous sandstone	0.6	25	0.24	26.42	4.3	26	2
Mudstone	5.22	14.3	0.2	25.69	7.2	24.4	4.21
Argillaceous sandstone	1.5	24	0.24	26.42	4.3	24	2
Fine sandstone	3.4	35.25	0.2	26.73	16.7	28.3	10.86
Mudstone	0.6	14.3	0.22	26.73	7.2	24.4	4.21
Fine sandstone	3.7	35.25	0.2	25.73	7.7	26.3	10.86
Mudstone	1	14.3	0.22	25.73	7.2	24.4	4.21
Fine sandstone	4.6	35.25	0.2	26.43	8.7	26.3	10.86
Mudstone	1.4	14.3	0.22	23.73	4.2	24.4	4.21
Carbonaceous mudstone	0.3	14.8	0.24	25.27	7.27	28	4.59
3#coal	5	6.3	0.33	18.3	3.2	32.4	2.33
Mudstone	1.14	14.3	0.22	25.73	7.2	24.4	4.21
Fine sandstone	0.9	35.25	0.2	26.73	16.7	30.3	10.86
Mudstone	5.73	14.3	0.22	25.73	7.2	24.4	4.21
Fine sandstone	2.23	35.25	0.2	26.73	16.7	30.3	10.86

The distance from the left boundary to the right boundary in the numerical computation model is 30 m of the coal seam at the 3505 working face, 5.5 m of return air lane at the 3505 working face, 1.5 m of a soft-moulded concrete pre-cast wall, 5.7 m of transport lane at 3503 after expanding the gangway, and 30 m of the coal seam at the 3503 working face. For the respective upper and lower boundaries, the rock layer of the top slab is the 11th mudstone, and the rock layer of the bottom slab of the roadway is 4th sandstone, and a total length of 135 m of the working face is simulated to be mined back, and the roadway to be dug is simulated to exhibit the same length. The whole model exhibits a fixed boundary except for the upper component which exhibits an applied uniform vertical stress of 5.5 MPa.

#### (2) Monitoring point arrangement

**Figure 13:**
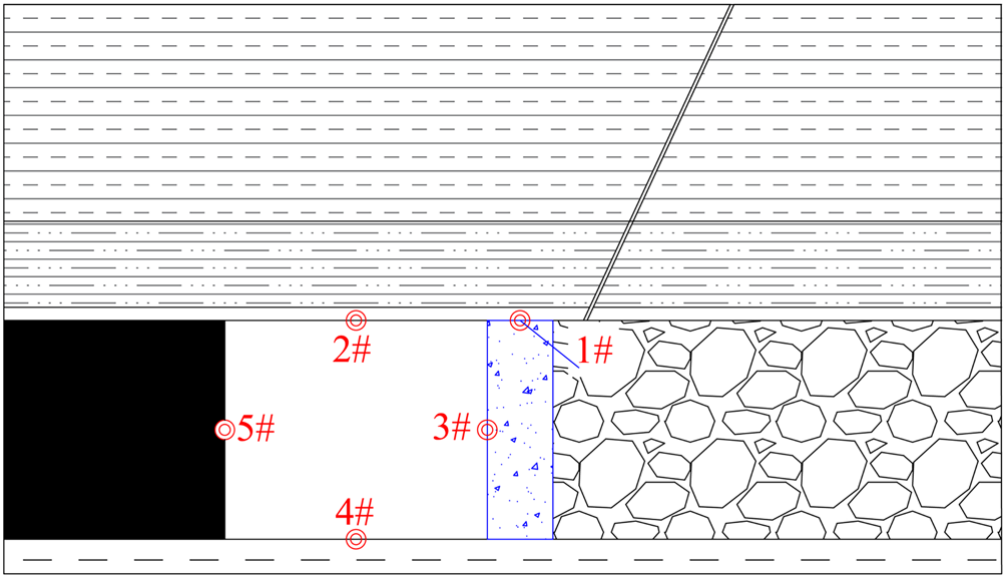
Distribution of monitoring points.

In the numerical simulations, five monitoring points are arranged (Nos. 1–5). Among them, monitoring points 1 monitor the stress changes in the roof and floor plate of the diaphragm wall in the flexible mould, and monitoring points 2–5 monitor the peripheral rock transport in the 3505 return air roadway. The distribution of monitoring points is depicted in Fig. 13.

The monitoring was divided into two phases:1) The first phase monitored the top stresses in the flexi-moulded pre-cast walls under cut-top and uncut-top conditions during the mining of the 3503 working face.2) In the second stage, the force on the roof and floor of the return air roadway in 3505 working face under the condition of roof cutting and non-roof cutting and the movement and change law of surrounding rock of the roadway are monitored.

### Numerical simulation of pre-cast walls after mining

**Figure 14:**
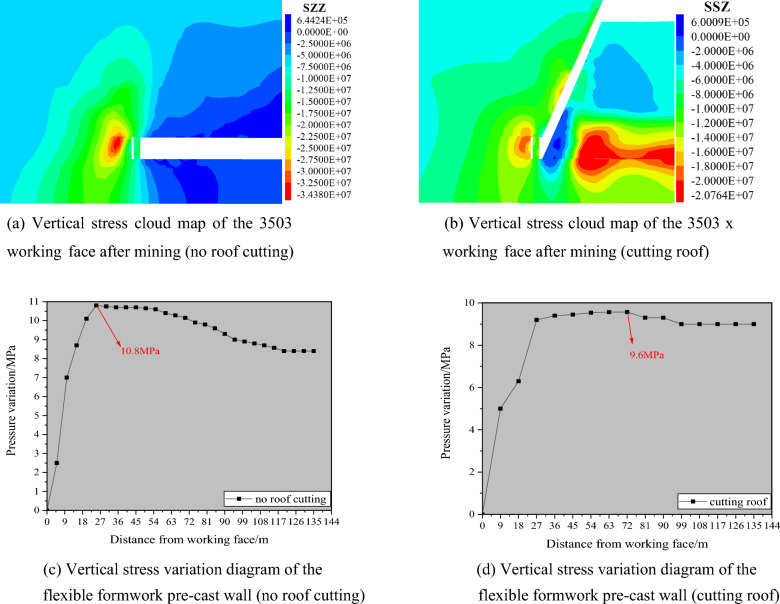
Numerical simulation analysis of post-workover mining. (**a**) Vertical stress cloud map of the 3503 working face after mining (no roof cutting), (**b**) vertical stress cloud map of the 3503 working face after mining (cutting roof), (**c**) vertical stress variation diagram of the flexible formwork pre-cast wall (no roof cutting), (**d**) vertical stress variation diagram of the flexible formwork pre-cast wall (cutting roof).

**Figure 15:**
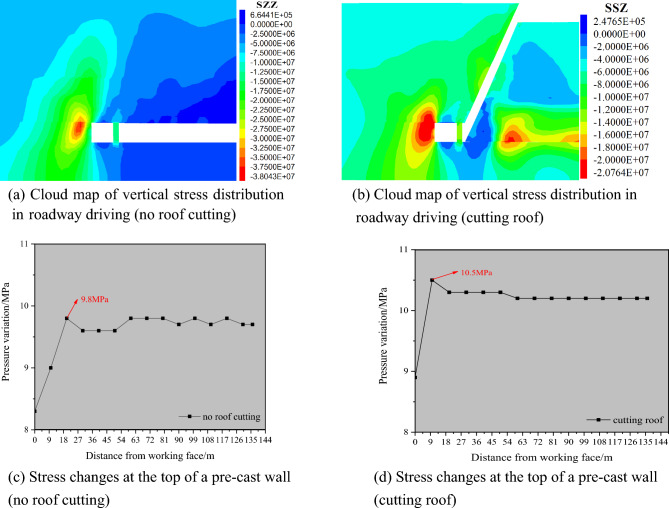
Cloud map of vertical stress distribution in roadway driving. (**a**) Cloud map of vertical stress distribution in roadway driving (no roof cutting), (**b**) cloud map of vertical stress distribution in roadway driving (cutting roof), (**c**) stress changes at the top of a pre-cast wall(no roof cutting), (**d**) stress changes at the top of a pre-cast wall (cutting roof).

Figures 14 and 15 depict the cloud diagrams of vertical stress distribution and the vertical stress change at the top of the flexible formwork pre-cast wall after mining back in the 3503 working face under the condition of no roof cutting and roof cutting, respectively.

From the graphical analysis, it can be observed that the maximum vertical stress at the top of the wall without cutting the top is 10.8 MPa, and that the maximum vertical stress at the roof of the wall with cutting the roof is 9.6 MPa. The maximum stress in the solid coal under the roof-cutting condition was reduced by 45% compared to the maximum stress in the solid coal without the roof-cutting condition. The overall force on the wall in the non-cutting roof condition increases and then decreases smoothly, whereas the overall force on the wall in the roof-cutting condition increases and subsequently smoothes out. The maximum vertical stresses during mining are mainly concentrated within the solid coal.

The comprehensive analysis results indicate that the non-coal pillar mining technology of flexible formwork pre-cast wall driving roadway along the goaf combined with advanced roof cutting and roadway protection technology can effectively reduce the stress of the wall and is beneficial to the roof and wall maintenance when driving roadway along the wall.

### Numerical simulation of roadway driving along the wall

#### (1) The influence of roof cutting on the vertical stress of the surrounding rock of roadway driving along the wall

Figure 15 exhibits the cloud picture of vertical stress distribution after driving in the return air roadway of the 3505 working face without roof cutting. From the graphical analysis, it can be observed that the maximum stress at the top of the pre-cast wall without cutting the roof is approximately 9.8 MPa; the maximum stress on the roof of the roadway is approximately 2 MPa; the maximum stress in the bottom plate is approximately 0.6 MPa; and the maximum stress of the coal side is approximately 1.3 MPa. The coal wall side stress gradually increases towards the solid coal, and the stress concentration range is large, attaining a stress peak of approximately 38 MPa. It can be observed that the maximum stress at the top of the pre-cast wall at the time of cutting the top is approximately 10.5 MPa; the maximum stress on the roof of the roadway is approximately 1.3 MPa; the stress in the bottom plate is considerably small (maximum value; approximately 0.8 MPa); and the maximum stress of coal side is approximately 16 MPa. Coal wall side stress gradually increases towards the solid coal, and the stress concentration range is small, and attains the peak stress value of approximately 22 MPa.

From the analysis depicted in Fig. 15, it can be observed that the vertical stress of the surrounding rock of the roadway driven along the goaf is apparently reduced after the roof cutting technology is adopted. The range of the stress concentrations on the solid coal side increased, but the overall stress was significantly reduced. This observation indicates that the use of cutting top treatment after the pre-cast wall in the transport lane of the 3503 working face can effectively reduce the vertical stress of the surrounding rock in the 3503 return lane, which is conducive to the maintenance and use of the roadway.

#### (2) The influence of roof cutting on the surrounding rock displacement of roadway driving along the wall

**Figure 16:**
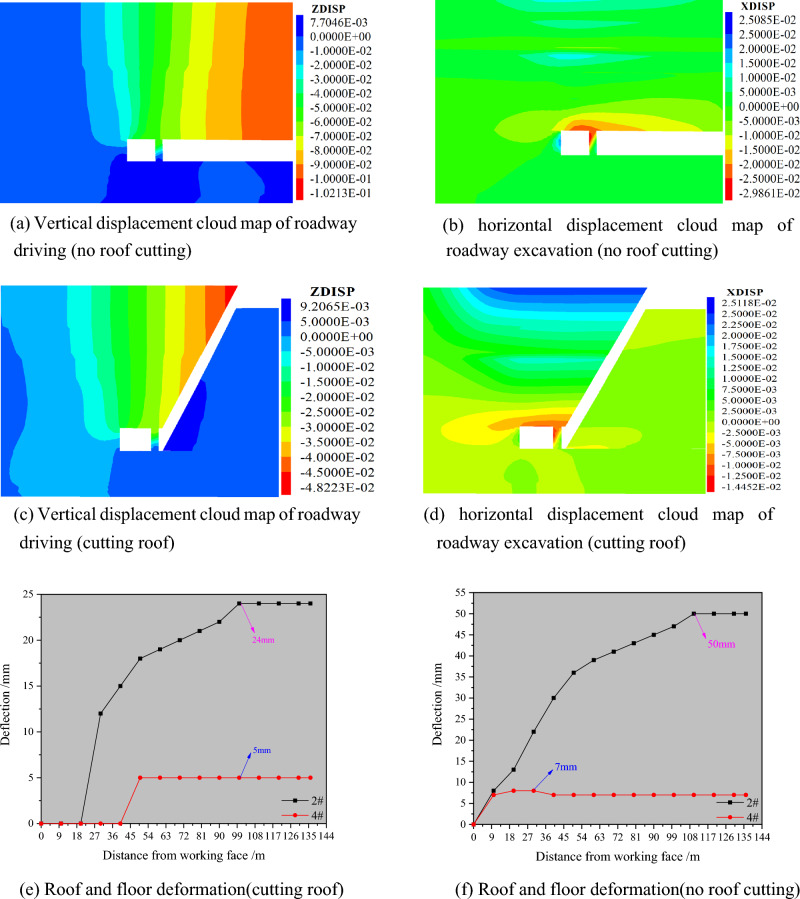

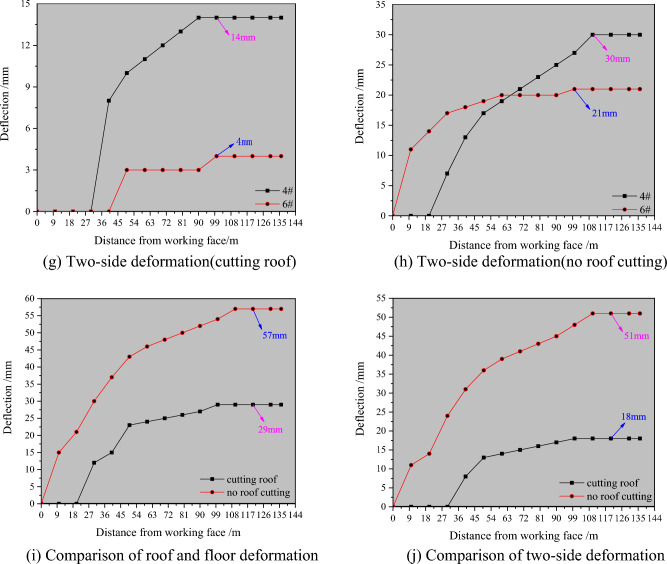
Displacement analysis of surrounding rock in roadway driving. (**a**) Vertical displacement cloud map of roadway driving (no roof cutting), (**b**) horizontal displacement cloud map of roadway excavation (no roof cutting), (**c**) vertical displacement cloud map of roadway driving (cutting roof), (**d**) horizontal displacement cloud map of roadway excavation (cutting roof), (**e**) roof and floor deformation(cutting roof), (**f**) roof and floor deformation (no roof cutting), (**g**) two-side deformation(cutting roof), (**h**) two-side deformation(no roof cutting), (**i**) comparison of roof and floor deformation, (**j**) comparison of two-side deformation.

Figure 16 presents the analysis diagram of surrounding rock deformation and displacement after driving a roadway in the 3505 return air roadway. According to the graph analysis, the maximum subsidence of the roadway roof is 50 mm, that of the floor is 7 mm, and that of the roof and floor is 57 mm without roof cutting. The maximum horizontal displacement on the side of the coal wall of the roadway is 21 mm, the maximum horizontal displacement on the side of the flexible wall is 30 mm, and the maximum displacement of the two sides is 51 mm. According to the graph analysis, the maximum subsidence values of the roadway roof, floor, and roof and floor are 24 mm, 5 mm, and 29 mm, respectively. The maximum horizontal displacement of the coal wall side of the roadway is approximately 4 mm, the maximum horizontal displacement of the flexible wall side is 14 mm, and the maximum displacement of the two sides is 18 mm.

From the analysis depicted in Fig. 16, it can be observed that under the roof cutting condition, the proximity of the roof and floor of the roadway is 49% less than that without roof cutting, and that of the two sides is 65% less than that without roof cutting. This observation can be rationalized as follows: the roof cutting technology is not performed, there is a lateral roof cantilever beam on the roof of the goaf, and the 3505 return air roadway and flexible formwork wall is affected by the cantilever structure, leading to crucial deformation and shear failure of the roadway and wall. After the roof-cutting process is conducted, the lateral cantilever beam is cut off, the stress of the roadway roof is reduced, the stress concentration area of the coal side becomes smaller and the stress value is also significantly reduced, and the roadway deformation of 3505 return air roadway is apparently reduced. Thus, it can be observed that the industrial practice pertaining to the non-pillar mining technology of the flexible formwork pre-cast wall driving roadway along goaf should be conducted in combination with advanced roof-cutting technology.

## Scheme design of roadway driving along goaf with a flexible formwork pre-cast wall

### Basic lane support

**Figure 17:**
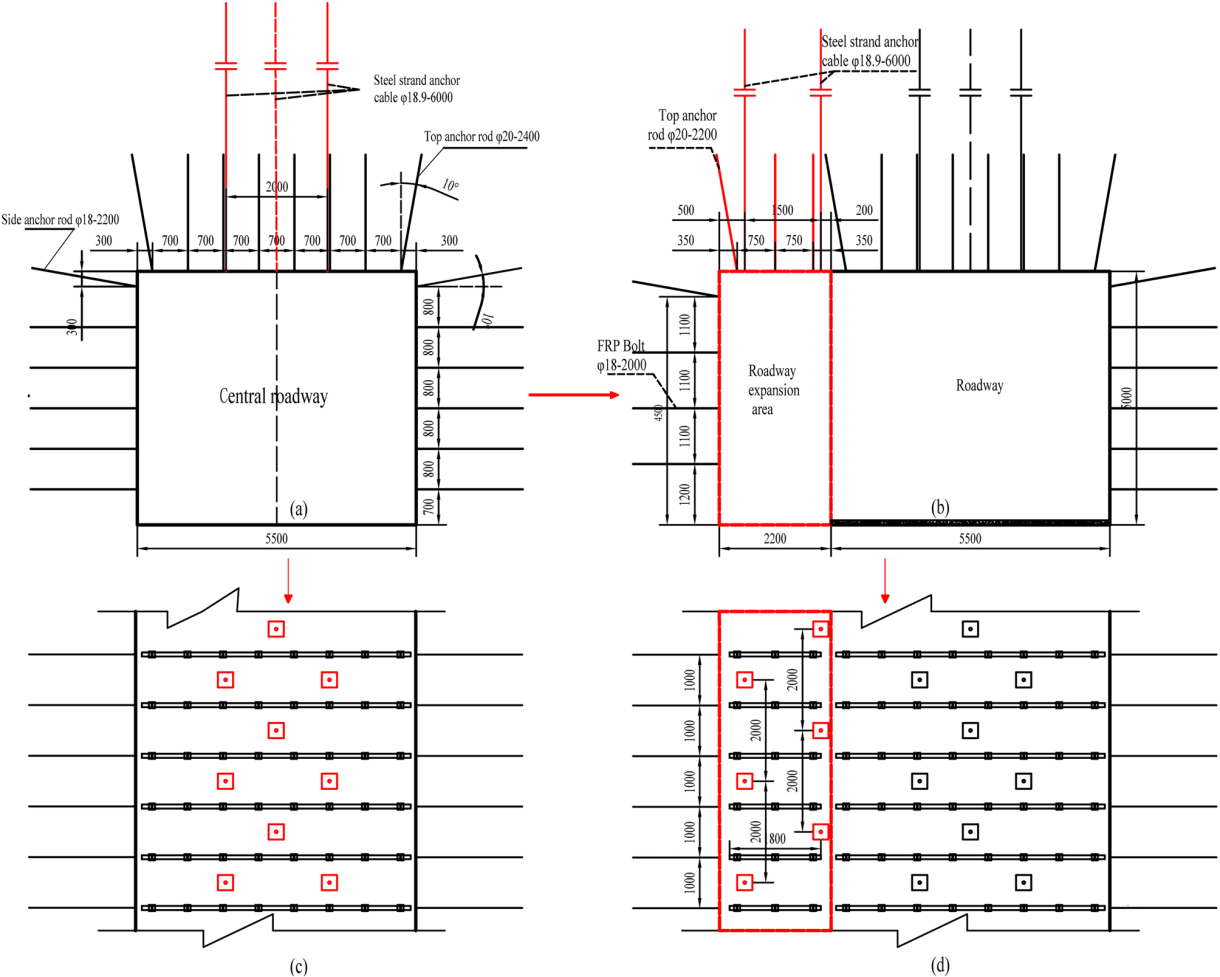
Basic support and expansion support for the roadway.

The 3503 working face transport roadway is arranged along the bottom plate of the No.3 coal seam, the section form is a rectangular section, the digging size is 5.5 m in width and 5.0 m in height, and the area of the digging section is 27.5 m^2^. The 3503 working face transport roadway is basically supported by anchor rods + warp and weft nets + steel ladder beams + anchor ropes, and the basic support cross-section is illustrated in Fig. 17a, c.

Figure 17 indicates that φ20 × 2400 mm high-strength rebar anchors are utilized in the roof plate of the roadway, with a spacing of 700 mm and a row spacing of 1000 mm, and that eight anchors are uniformly installed in each row, with six anchors in the middle arranged vertically on the roof plate, and two anchors at an angle of 10° with the vertical straight line pointing to the outer side of the roadway, 300 mm away from the coal side; and the top plate of the roadway utilizes φ18.9 × 6000 mm anchor ropes with a spacing of 2000 mm and a row spacing of 1000 mm, adopting a five-flower arrangement. The metal mesh is a welded 10# lead wire; the welded and processed double-reinforced double-beam steel ladder beam utilizes φ14 round steel. The roadway side utilizes Ф18 × 2200 mm high-strength rebar anchors with a spacing of 800 mm and a row spacing of 1000 mm. 12 anchors are arranged in each row; the upper two anchors are at an angle of 10° to the horizontal line, pointing to the top plate, with a distance of 300 mm from the top plate, and the following ten anchors are arranged vertically on the roof of the coal gangs. The metal mesh is welded using No. 10 lead wire with a characteristic mesh hole size of 50 mm and a mesh width of 1050 mm; the welded and processed double-reinforced double-beam steel ladder beam utilizes φ14 round steel.

### Advance expansion support

1. Expanding position: starting from the cutting position of the 3503 working face, the experiment of driving roadway along goaf with a flexible form pre-cast wall is performed along the side of the coal wall of the 3505 working face, and the subsequent wall construction is determined according to the 900 m test effect.
2. Expansion section: the width of the transportation roadway along the coal wall side of the 3503 working face is 2200 mm, and the height of the expansion is 5000 mm. The support cross-section after expansion is depicted in Fig. 17(b,d). The specific roof and side support are as follows:

- Roof support
  Use φ20 × 2200 mm high-strength rebar anchor rods and φ14mm round steel reinforcement beams with mesh. 750 mm spacing and 1000 mm row spacing are applied; 3 rods are evenly placed in each row; 2 rods are placed in the middle at an angle of 90° to the roof plate; and the outer anchors, which are tilted outward at an angle of 10°, are arranged at a distance of 35 0 mm from the coal gangs. Use a Ф18.90 × 6000 mm anchor cable, staggered arrangement of two rows of anchor cable in the gang expansion area: one row of anchor cable is 500 mm away from the coal gang (row spacing; 2000 mm); the other row of anchor ropes is 2000 mm away from the coal gang with a 2000 mm row spacing.
- Two sides of support
  Use φ18 × 2000 mm FRP anchor rods, four rods in each row (spacing: 1100 mm and row spacing: 1000 mm); the one above the roadway gang is 500 mm away from the top plate and is set at an upward inclination of 10°, and the following three rods are 1200 mm away from the roadway bottom plate and are set in a horizontal direction.

### Parametric design of the flexible formwork wall

**Figure 18:**
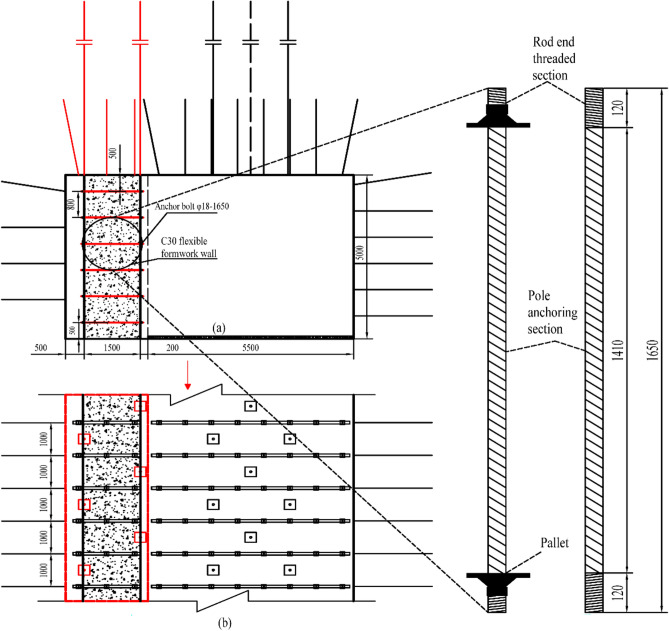
Flexible formwork wall parameters and design section.

To facilitate the support of flexible formwork, a 500 mm deformation space was reserved in the coal wall measurement of the 3505 working face; the design strength of concrete was determined to be C30 by theoretical calculations and numerical simulation analysis; the textile structural flexible formwork was developed in-house and has a height of 5000 mm, a width of 1500 mm, and a length of 3000 mm; each row of anchor bolts is combined as a whole on both sides of the wall using φ14mm round steel welded reinforcement beams, which are 80 mm wide and 4400 mm long; the spacing of the limit holes in the reinforced beams is 800 mm, matching the anchor bolt arrangement; an anchor bolt φ18 × 1650 mm high strength rebar and rebar grade not less than No. 335 were applied; both ends of the rod are provided with wire fasteners, and the length of each end of the wire fastener is not less than 100 mm; each end of the rod is equipped with a set of high-strength pallets, centring spherical pads, and nylon washers, and the pallets comprise 120 mm × 120 mm × 8 mm arched high-strength trays; 3 days after the flexible formwork wall has been moulded, apply a pre-tensioning force to the anchor bolts, with a pre-tensioning torque of not less than 150 N m; anchor bolts are six in each row with an inter-row spacing of 800 × 750 mm. The support section and anchor bolt are shown in Fig. 18.

### Cutting roof process

#### (1) Types and specifications of explosives

The roof-cutting process is carried out by blasting with Class III coal mine permitted emulsion explosives and are detonated with No. 8 ordinary detonators. The specification of the explosives roll is Φ 35 × 200 mm, and each roll weights 0.2 kg.

#### (2) Borehole arrangement

Along the transportation roadway of 3503 working face, a group of blastholes are arranged every 4 m, every three blastholes as a group, and the distance between blastholes in each group is 2 m. The blasthole is arranged along the roof at 0.5 m of the outer side (flexible formwork wall) of the transport roadway (considering the position of roof anchor and anchor cable, which can be adjusted within the range of ± 0.2 m). The axis direction of the blasthole is parallel to the direction of the transport roadway, and the elevation angle is 65°(the angle between the blasthole and the roof, pointing to the goaf of the working face), the length of the blasthole is 17.6 m, and the diameter of the blasthole is 75 mm.

#### (3) Time and manner of blasting of blast holes

When the distance between the first hole of each group of holes and the coal wall of the 3503 working face is within 5–20 m, the group of holes can be loaded and discharged. Discharge the gun using a BF-200 type detonator for group loading and blasting. A crop of gun lines is selected using the "local parallel, the overall series" form of development; each detonation requirements of up to three holes, and the provisions of the shift loaded holes should be blasting the same shift.

### Engineering practice

#### Industrial tests

**Figure 19:**
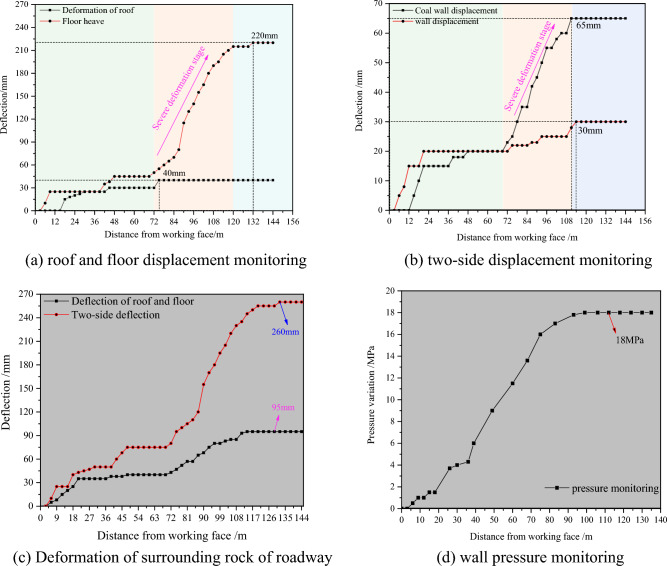
Monitoring curve. (**a**) Roof and floor displacement monitoring, (**b**) two-side displacement monitoring, (**c**) deformation of surrounding rock of roadway, (**d**) wall pressure monitoring.

According to the design scheme of driving roadway along goaf with a flexible formwork pre-cast wall, an industrial test was performed in the 3503 working face of Wangzhuang Coal Mine. when driving roadway in the 3505 working face (the support mode of roadway roof and coal wall is the same as 3503), the surrounding rock displacement of roadway (including roof subsidence, bottom sinking, and two wall approaching) and flexible mold wall pressure monitoring are performed synchronously. According to the monitoring data, the monitoring curve can be obtained as illustrated in Fig. 19.

Figure 19a indicates that with the return air roadway driving along the wall in the 3505 working face, the roadway roof deformation increases, but the overall range is small. When the lagging digging surface exceeds 72 m, the roadway roof subsidence increases rapidly, and finally tends to stabilise after 115 m, at which time the roof subsidence is 220 mm; the amount of change in the bottom drum of the roadway is small throughout the excavation stage, with maximum stability of 40 mm. Figure 19b, c indicates that with the return air roadway driving along the wall of the 3505 working face, the variation law of the approach of the two sides is similar to that of the roof and floor, but the approach of the two sides is generally less than that of the roof and floor, in which the maximum approach of the wall is 65 mm, the maximum approach of the coal wall is 30 mm, and the approach of the two sides of the roadway is basically stable after 110 m behind the heading face. Figure 19d indicates that the overall pressure deformation of the wall left along the goaf can be divided into three stages: in the first stage, the wall pressure increases slowly within the range of 0 mm 35 m of the lagging working face, from 0 to 4 MPa; and in the second stage, the wall pressure increases sharply from 4 to 18 MPa within 35–100 m of the lagging workface; in the third stage, the wall pressure is basically stabilised at about 18 MPa after 100 m of lagging workface.

From the monitoring results of the industrial test section, it can be observed that the overall deformation of the top and bottom plates and the two gangs of the roadway is relatively small, and it can be utilized as a return-airway for the 3505 back-mining face.

#### Industrial applications

**Figure 20:**
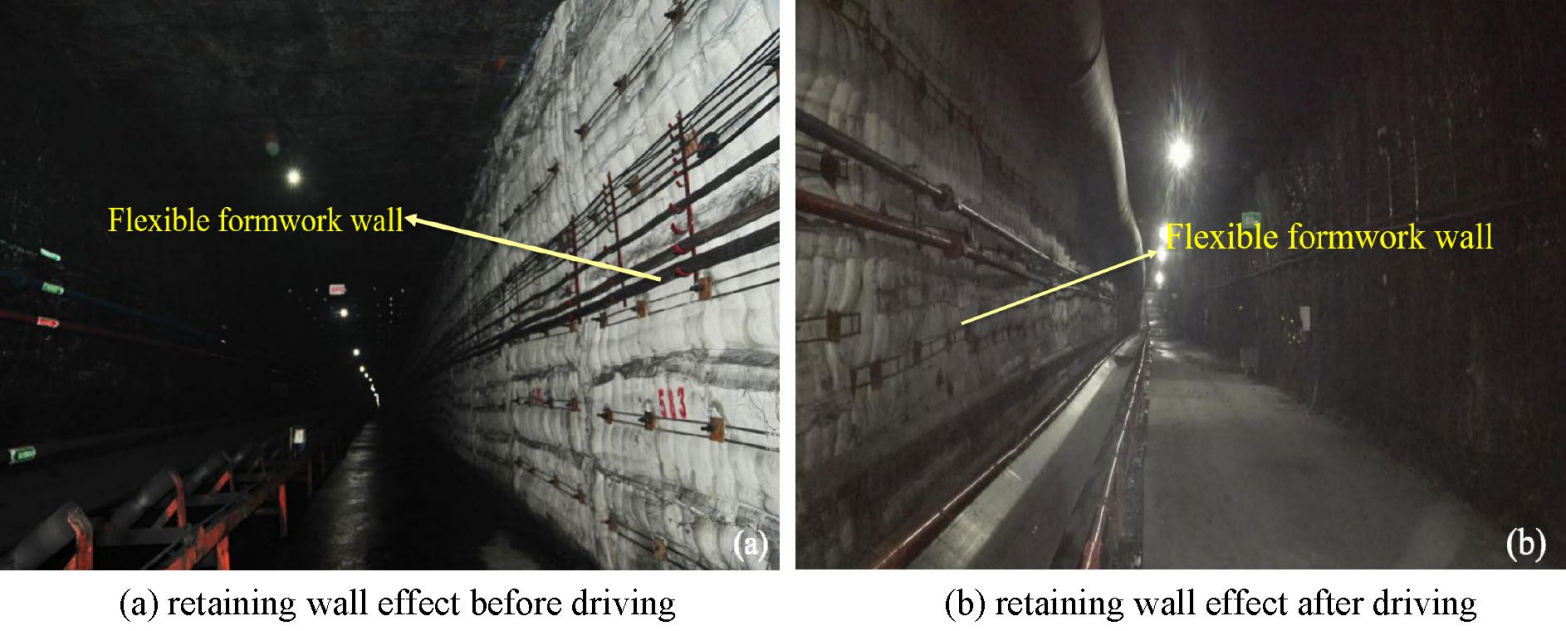
Effect diagram of industrial application. (**a**) Retaining wall effect before driving, (**b**) retaining wall effect after driving.

The industrial test monitoring results indicate that the design of expanding support and wall parameters of roadway expansion and wall parameters are rational, and that the overall deformation of roadway after driving is small; therefore, this technology can be applied to the non-pillar mining of transportation roadway in the 3503 working face. Because the technology of the retaining wall along goaf at a 5 m mining height is applied for the first time, the surrounding rock deformation and wall pressure of the roadway are continuously monitored in the industrial application stage; thus, support optimization can be conducted later. The specific industrial application effect is as follows:

1. Application effects
  The working face transport roadway flexible formwork pre-cast wall driving roadway along the goaf has experienced three stages of mining influence: The first stage is the leading and lagging dynamic pressure in the mining stage of the 3503 working face; the second stage is the leading and lagging dynamic pressure of the 3505 working face along the wall; and the third stage is the leading dynamic pressure in the mining stage of the 3505 working face. The application effect of the flexible formwork pre-cast wall driving roadway along goaf in the 3503 working face is depicted in Fig. 20.

From the analysis, it can be observed that the effect of the pre-poured wall before driving roadway in Fig. 13a belongs to the first stage of mining influence. The monitoring of the displacement of the roadway perimeter rock and the pressure of the flexible formwork walls belongs to phase 2 of the mining impact; Fig. 13b indicates that the effect of the pre-poured wall after driving roadway belongs to the third stage of mining influence. The effect of on-site pre-cast wall and the monitoring results of industrial test indicate that the application of the flexible mold pre-cast wall along goaf driving technology in the 3503 working face of Wangzhuang Coal Mine exerts a satisfactory effect in the whole stage. It solves the technical problems of large mining height face in thick coal seam, such as high roadway, high support pressure beside roadway, low early strength of support body, easy to be damaged by compression, difficult to support roof effectively, severe mine pressure in large mining height working face, and difficult maintenance beside roadway. The proposed method provides a novel technical choice for how to recover a coal pillar in a 20 m section, 5 m high mining face and improve the recovery rate of coal resources and roadway driving speed, and can be widely utilized in coal seam mining in this mine and even in the two cities.

## Conclusion

Based on the research background of the 3503 working face with a 5 m mining height in Wangzhuang Coal Mine, this paper proposes an innovative non-pillar mining technology: "flexible formwork pre-pouring wall driving roadway along goaf". This technology can provide a novel choice for the technical problems occasioned by the high height of the roadway and the high pressure of roadside support in the goaf-side roadway of thick coal seam. Using a laboratory test, theoretical analysis, numerical simulation analysis, and industrial practice, the following conclusions and results are obtained herein.

1. Flexible formwork concrete walls exhibit high initial bracing strength. The 0.5 day uniaxial compressive strength of the flexible formwork concrete was 0.8 MPa, the 1 day uniaxial compressive strength was increased by 30% relative to ordinary concrete, and the 28 days uniaxial compressive strength was increased by 20%.
2. Through theoretical analysis and numerical calculations, the final decision was arrived at: use C30 strength, 1.5 m width flexible formwork concrete walls to perform numerical simulation and industrial test studies.
3. According to the theoretical calculation results and the geological conditions of the 3503 working face in the Wangzhuang coal mine, numerical simulation of flexible formwork pre-cast wall along the hollow digging roadway was conducted. The simulation analyses the stress distribution law of the flexible formwork concrete wall under the condition of no top-cutting and roof cutting after mining back in the 3503 working face, and the stress and deformation law of the roadway enclosure under the condition of no roof cutting and roof cutting after digging back to the return roadway in the 3505 working face is determined.
4. Combined with the theoretical calculation and numerical simulation results, the scheme design and industrial practice of roadway driving along goaf with a flexible formwork pre-cast wall are performed. The results indicate that the application of a flexible formwork pre-cast wall driving roadway along goaf in the 3503 working face of Wangzhuang Coal Mine exerts a favourable effect in the whole stage, which solves the technical problems such as high roadway, high roadside support pressure, the low early strength of the support body which is easily damaged by pressure and difficult to effectively support the roof plate, and the difficulty of roadside maintenance due to the drastic manifestation of mining pressure in the face of large mining height.
5. The non-pillar mining technology of the flexible formwork pre-cast wall driving roadway along goaf has recovered the coal pillar that exhibits a 20 m section and 5 m height mining face in Wangzhuang Coal Mine. As a result, the recovery rate of coal resources and the driving speed of the roadway was significantly improved. The aforementioned technology can be popularised and applied in this mine and even in the mining of 15# large-height coal seams in the two cities.
